# Dissecting ventricular pseudoaneurysm after perimyocarditis—a case report

**DOI:** 10.1186/s13019-015-0373-z

**Published:** 2015-11-06

**Authors:** Rickard P.F. Lindblom, Ulrica Alström, Vitas Zemgulis

**Affiliations:** Department of Cardiothoracic Surgery and Anesthesia, Uppsala University Hospital, 751 85 Uppsala, Sweden

**Keywords:** Perimyocarditis, Pseudoaneurysm, Echocardiogram

## Abstract

**Background:**

The current case describes the fast development of a pseudoaneurysm in a patient that presented with signs of systemic inflammation and generally deranged blood work.

**Case Presentation:**

The pseudoaneurysm appeared within one week of disease onset. The anatomic extent of the pseudoaneurysm was unusual, as it dissected intramurally beneath the septum, inferior to the right ventricle and had effect on the RV filling. The etiology could not be definitely defined, since in adults the most common cause for pseudoaneurysm development is recent myocardial infarction, but in this patient the coronary arteries were healthy. Instead it could have been a consequence of an aggressive perimyocarditis.

**Conclusions:**

Due to the unpredictable nature of pseudoaneurysms we advocate early contact with a center with cardiothoracic surgery expertise for rapid surgical intervention.

## Background

Pseudoaneurysms differ from aneurysms in that they lack the components of the ordinary myocardial wall and are only contained by fibrous membrane [1], thus the significant risk of rupture, up to 45 % [2]. Transmural myocardial infarction is the most common etiology behind development of pseudoaneurysms, but they can also occur after trauma, infection and surgery [3–5].

## Case presentation

A 48 year old man presented in a rural hospital with nausea, vomiting, diarrhea, fever and high sitting abdominal pains radiating towards the left shoulder. He had a previous medical history of unspecified collagenosis and had since the age of 12 been hospitalized on eight occasions with diffuse rheumatic symptoms that had been treated with cures of steroids and antibiotics. He was in good physical shape without any symptoms of heart disease, without any medications, led an active life and had recently performed an ergometry test that was normal.

Upon presentation there were general ST-elevations on the ECG, suggestive of perimyocarditis. He had elevated levels of white blood cell count (24.1, ref 3.5–8.8), C-reactive protein (152, ref <5), creatinine (165, ref 60–105), hepatic transaminases (ASAT/ALAT, 3.1/3.36, ref <0.75/<1.1) and troponin I (15, ref <0.07). Blood pressure was 100/70 and there was jugular vein distension. A CT scan was performed; this revealed a significant amount of pericardial fluid, right sided pleural effusion, enlarged mediastinal lymph nodes, oedema around the pancreas and fluid around the liver. A transthoracic echocardiogram confirmed the presence of pericardial effusion with significant hemodynamic compromise, but no other significant findings.

**Fig. 1:**
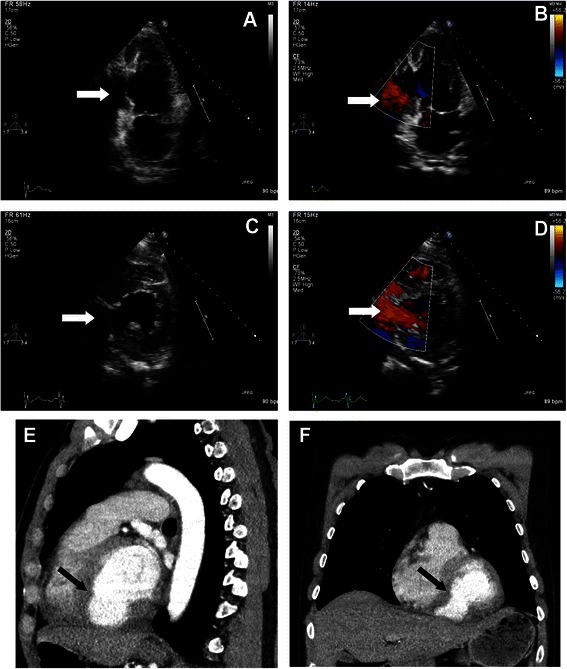
Preoperative TTE and CT. A preoperative transthoracic echocardiogram showing the left ventricle and the posteroinferior cavity of the pseudoaneurysm (**a**), with turbulent flow inside (**b**). A TTE cross-sectional view showing the large ostium to the cavity (**c**) and (**d**). A preoperative CT exam determines that the large posteroinferior cavity extends beneath the septum (**e**) and (**f**). The white (A–D) and black (E and F) point to the pseudoaneurysm

This warranted urgent transfer to a county hospital where antibiotics (Cefotaxime) were started. In the next days a new echocardiogram was performed which identified a 2.7 × 3.3 cm large pseudoaneurysm in the posteroinferior wall of the LV (Fig. 1a–d). The ostium of the pseudoaneurysm was localized close to the posterior leaflet of the mitral valve, but the valve was patent. Moreover, the pericardial effusion had diminished. A new CT-scan was performed which demonstrated a rupture in the ventricular wall with an ostium of 2–3 cm (Fig. 1e–f). The cavity dissected in the muscular mass beneath the septum and affected the filling of the RV. A coronary angiogram was performed, which surprisingly showed no coronary pathology, after which the patient was moved to the department of cardiothoracic surgery. The operation took place 13 days after initial hospitalization.

**Fig. 2:**
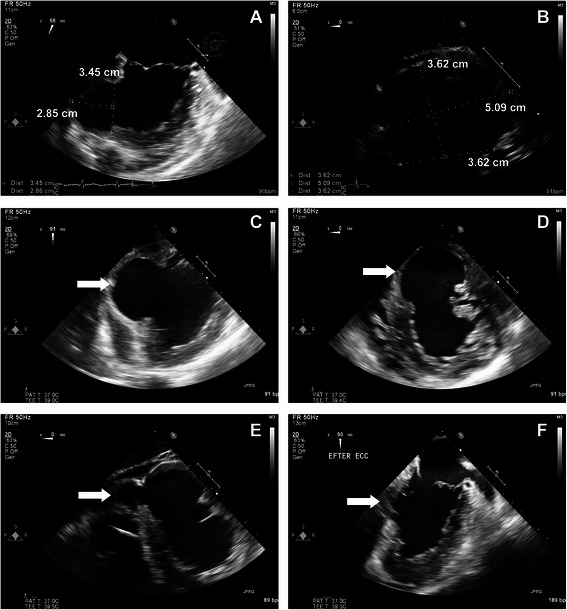
Peri-and postoperative TEE. Perioperative transesophageal echocardiogram demonstrates that the cavity is almost as large as that of the LV, and probably has increased in size from the previous TTE (**a**–**d**), white arrow points to the pseudoaneurysm. The cavity dissects beneath the septum and under the right ventricle (**e**), white arrow points to the part beneath the RV. TEE at the end of surgery shows that the cavity is largely obliterated (**f**), the white arrow points to where the ostium was

**Fig. 3:**
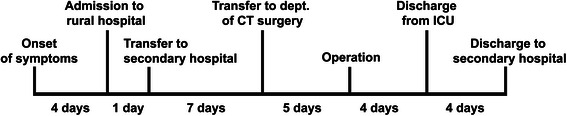
Timeline depicting the disease course. A schematic timeline depicting the disease course and events

The patient was cannulated in the femoral vessels and sternotomy performed on full bypass. The pericardium was opened and a substantial volume of dark red fluid with fibrin clots was released under pressure. The posterodiaphragmal wall of the LV consisted of a hematoma with a large amount of blood clots. The hematoma, i.e. the epicardium that contained the pseudoaneurysm was opened; the aneurysmal sac disrupted the distal part of the septum and dissected intramyocardially into the RV and out to the epicardium (Fig. 2a–e). A 5 × 5.5 cm cardiovascular patch was anchored to the septum with Teflon pledgets, with the sutures passing through the dissection space beneath the RV and out to the epicardium on either side of the pseudoaneurysm edges through a second Teflon strand forming a three-layer sandwich construction. The edges of the pseudoaneurysm were adapted using a second patch and a running suture over the first patch. There was also a thinning of the RV where the aneurysmal sac had dissected. This was stabilized using new Teflon strands and interrupted sutures. Obliteration of the cavity was confirmed by TEE (Fig. 2f). Weaning from cardiopulmonary bypass was done with inotropic support and was complemented with a percutaneously placed IABP which reduced the needs of amines. The IABP was removed on the third postoperative day. Eight days postoperatively the patient was discharged to the home clinic in good condition (Fig. 3). Postoperative TTE revealed a moderately depressed LV function, hypo/akinesia of the septum and the inferior wall by the patch, and a mild TR but no MR. The perioperative biopsy of the tissue in the pseudoaneurysm revealed the presence of fibrin and low numbers of eosinophils, suggestive of infarcted myocardium. No amyloid or excessive inflammation was seen. The patient was negative for CMV, EBV and legionella antigen and no bacteria were identified in the cultures. At follow up 6 months after surgery the patient was in good clinical condition.

## Conclusions

The explanation behind the development of the current pseudoaneurysm remains a puzzle as there was no coronary pathology and no site specific infection. We believe it was a consequence of the perimyocarditis, which in general is a benign condition [6], where the current case is clearly an exception. Cases of peri-/myocarditis that have led to ventricular pseudoaneurysm development have been described in children [7, 8] and in conjunction with tuberculosis [9]. Another described etiology behind pseudoaneurysm development is severe endocarditis, with abscess formation [10]. However in the above mentioned cases there has been verified bacterial infections, most often *S.aureus*. In the current case there was a general systemic inflammation, not a site specific cardiac infection. Furthermore no cultures from the patient grew bacteria and 16sRNA analysis showed no presence of bacterial RNA. An exceedingly scarce [11] cause behind pseudoaneurysm formation is infiltrating tumors, but this was ruled out as the PAD showed no signs of malignancy. Another interesting aspect of the current case is that the pseudoaneurysm dissected intramyocardially beneath the septum instead of rupturing. This undoubtedly saved the patient’s life, but illustrates how unpredictable pseudoaneurysms are. In the light of this, we advocate early contact with a cardiothoracic surgery center for surgery with short delay.

## Consent

Written informed consent was given by the patient.

## Abbreviations

IABP: Intra-aortic balloon pump
LV: Left ventricle
MR: Mitral regurgitation
RV: Right ventricle
TEE: Trans-esophageal echocardiogram
TTE: Trans-thoracic echocardiogram
TR: Tricuspid regurgitation
